# mtDNA-STING pathway promotes necroptosis-dependent enterocyte injury in intestinal ischemia reperfusion

**DOI:** 10.1038/s41419-020-03239-6

**Published:** 2020-12-11

**Authors:** Xufei Zhang, Jie Wu, Qinjie Liu, Xuanheng Li, Sicheng Li, Jun Chen, Zhiwu Hong, Xiuwen Wu, Yun Zhao, Jianan Ren

**Affiliations:** 1 Research Institute of General Surgery, Jinling Hospital, Nanjing Medical University, Nanjing, 210002 PR China; 2 Research Center of Surgery, BenQ Medical Center, the Affiliated BenQ Hospital of Nanjing Medical University, Nanjing, 210021 PR China; 3 Research Institute of General Surgery, Jinling Hospital, Medical School of Nanjing University, Nanjing, 210002 PR China

**Keywords:** Necroptosis, Cell death and immune response

## Abstract

Intestinal ischemia reperfusion (I/R) injury is the important pathogenesis for acute intestinal barrier disruption. The STING signaling is associated with gut homeostasis and barrier integrity. However, the biological function and regulation of STING signaling in intestinal I/R injury are not yet fully understood. As the ligand of STING signaling, the mitochondrial DNA (mtDNA) has been found to be associated with necroptosis. It still remains unknown whether mtDNA-STING signaling triggers intestinal necroptosis in intestinal I/R injury. We found that circulating RIPK3 was significantly increased and had a positive correlation with markers of enterocyte injury in critically ill patients with intestinal injury. Moreover, the levels of circulating mtDNA were also associated with the levels of circulating RIPK3. To explore the relationship between mtDNA and intestinal necroptosis, mice were treated with the intraperitoneal injection of mtDNA, and necroptosis signaling was remarkably activated and the inhibition of necroptosis alleviated mtDNA-induced intestinal injury. Furthermore, STING knockout mice showed an alleviated intestinal necroptosis. In intestinal I/R injury, mtDNA was released from IECs and necroptosis was also triggered, companied with a significant decrease of RIPK3 in the intestine. STING knockout mice markedly attenuated intestinal necroptosis and intestinal I/R injury. Finally, we found that mtDNA-mediated STING signaling triggered necroptosis through synergistic IFN and TNF-α signaling in primary IECs. Our results indicated that mtDNA-STING signaling can contribute to intestinal I/R injury by promoting IEC necroptosis. STING-mediated both IFN and TNF-α signaling can trigger intestinal nercroptosis.

## Introduction

The intestinal ischemia reperfusion (I/R) injury is a life-threatening pathophysiological condition that commonly occurs in acute mesenteric ischemia, traumatic shock, hemorrhagic shock, sepsis, and some surgical procedures^1,2^. This process eventually leads to the disruption of the mucosal barrier integrity on account of severe mucosal injury and cell death. Moreover, the overwhelmed intestinal barrier is associated with increased intestinal permeability and facilitates the translocation of bacteria from the luminal environment to the circulation, resulting in systemic inflammatory response syndrome or multiple organ dysfunction syndrome^3^.

In addition, intestinal I/R injury also causes the massive release of damage-associated molecular patterns (DAMPs) and subsequently leads to severe inflammatory responses^4^. The mitochondrial DNA (mtDNA) is a potent DAMPs and can be released during the intestinal I/R process. Mounting evidence suggests that mtDNA regulates inflammation and contributes to disease pathogenesis through interaction with multiple signal pathways, including stimulator of interferon genes (STING)^5^. Our team has found that mtDNA originated from intestinal epithelial cells (IECs) exacerbates the inflammatory responses and gut barrier dysfunction during intestinal I/R injury^6^. Nevertheless, the function and explicit sensors of mtDNA in intestinal I/R injury have not yet been clarified.

The STING signaling has been shown to be a critical role in metabolic disorders, anti-tumor immunity, infectious and inflammatory diseases through the recognition of bacterial DNA or self DNA^7^. The mtDNA originated from damaged cells is also the potent ligand for STING signaling via cGAS (cyclic GMP-AMP synthase)^8^. The activation of STING pathway simultaneously triggers TANK-binding kinase 1 (TBK1), which induces the phosphorylation of both interferon regulatory factor 3 (IRF3) and NF-κB pathway, subsequently increasing the expression of type I interferon (IFN) and TNF-α^9^. In recent studies, STING has been revealed to play an emerging role in regulating gut homeostasis and barrier integrity^10,11^. However, it remains unclear how STING affects intestinal I/R injury. We have demonstrated that excessive activation of STING can disrupt the intestinal barrier in the murine cecal ligation perforation model^12^. Therefore, STING may be implicated in intestinal I/R injury.

Necroptosis, a programmed cell death mechanism, is driven by multiple death receptors, pattern recognizing receptors, and cytokine, and plays important roles in many inflammatory conditions and disease states^13^. This death was initially reported to be associated with receptor-interacting protein kinase 1 (RIPK1) and receptor-interacting protein kinase 3 (RIPK3). Upon stimulation, RIPK1 and RIPK3 are activated by autophosphorylation in the necrosome complex^14^. Subsequently, the mixed lineage kinase domain-like protein (MLKL) is recruited and phosphorylated by RIPK3, leading to MLKL oligomerization. Oligomerized MLKL is translocated onto the cell membrane, contributing to plasma membrane permeabilization and hence triggering necroptosis^15^. RIPK3 and MLKL are currently believed to be the major targets that mediate this form of death^14^. Despite the confirmed role of necroptosis in intestinal I/R injury^16^, how to regulate necroptosis in intestinal I/R injury remains unclear.

Recently, it is reported that the release of mtDNA can promote necroptosis signaling^17^. Therefore, we hypothesized that mtDNA-dependent STING pathway is involved in the pathogenesis of intestinal I/R injury through inducing IEC necroptosis. Here in this study, we observed that circulating RIPK3 was associated with intestinal injury, and there was a connection between circulating mtDNA and necroptosis. We further determined that mtDNA activated intestinal necroptosis contributing to the disruption of the intestinal barrier in intestinal I/R injury. Moreover, STING knockout alleviated mtDNA-induced necroptosis in the intestinal I/R model.

## Results

### Increased mtDNA is associated with necroptosis related to intestinal injury in patients with intra-abdominal infection

**Fig. 1:**
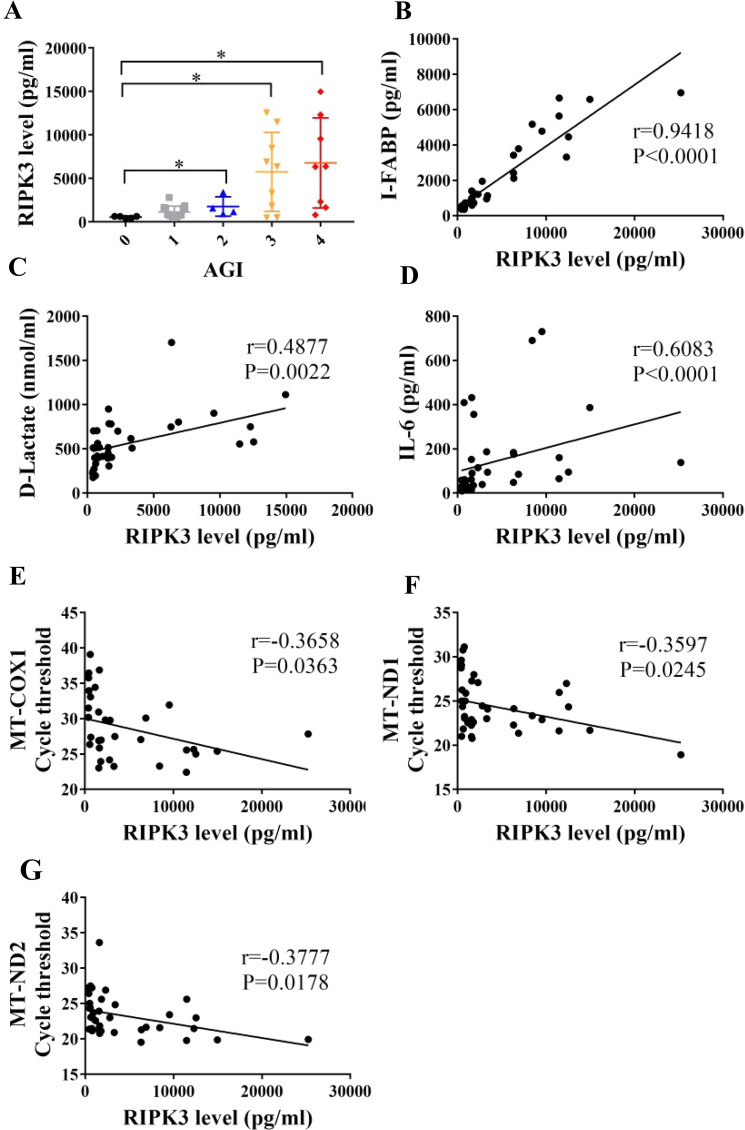
Elevated serum RIPK3 is associated with intestinal injury in critically ill patients. (**A**) Circulating RIPK3 in critically ill patients with 1–4 of acute gastrointestinal injury (AGI) scores and healthy control (AGI score 0) was analyzed through enzyme-linked immunosorbent assay (ELISA). (**B**–**D**) Serum I-FABP, D-lactate, and IL-6 were assessed by ELISA. (**E**–**G**) Correlation between mtDNA and circulating was performed. mtDNA was analyzed via quantitative real-time PCR analysis by amplifying three kinds of mtDNA primers. *P < 0.05, **P < 0.01 vs control group.

It has been shown that circulating RIPK3 levels were associated with poor outcomes during critical illness^18^. To explore the role of necroptosis in critically ill patients with intestinal injury, we firstly enrolled patients with intra-abdominal infection in the ICU and determined whether RIPK3 levels alter in the serum of these patients with intra-abdominal infection. Patients were divided into 4 groups according to the acute gastrointestinal injury (AGI) grading system and the group with AGI grade 0 was the healthy control. RIPK3 levels were elevated in critical patients with AGI grade II, III, and IV (Fig. 1a). Intestinal fatty acid-binding protein (I-FABP) and D-lactate, the circulating markers of enterocyte damage^19^, were also detected. In this study, we found that circulating RIPK3 levels had a significant correlation with circulating I-FABP and D-lactate levels (Fig. 1b, c), which points out the potential relationship between necroptosis and intestinal injury. Moreover, there was a positive correlation between serum RIPK3 and IL-6 (Fig. 1d). Therefore, the circulating RIPK3 levels could serve as a marker of intestinal injury and inflammation in critically ill patients.

Our previous studies revealed that the released mtDNA, as a DAMPs, induced gut barrier dysfunction^6^. It was also reported that mtDNA could serve as an initiator for necroptosis^17^. We then investigated the correlation between mtDNA and necroptosis. DNA was extracted from the serum of these patients and mtDNA levels were evaluated by amplifying three kinds of mtDNA sequences via qPCR. Of note, higher cycle thresholds represent lower levels of mtDNA. We found that circulating RIPK3 levels had a positive correlation with mtDNA concentration (Fig. 1e–g), indicating a strong connection between mtDNA and necroptosis.

### mtDNA contributes to necroptosis-dependent intestinal injury

**Fig. 2:**
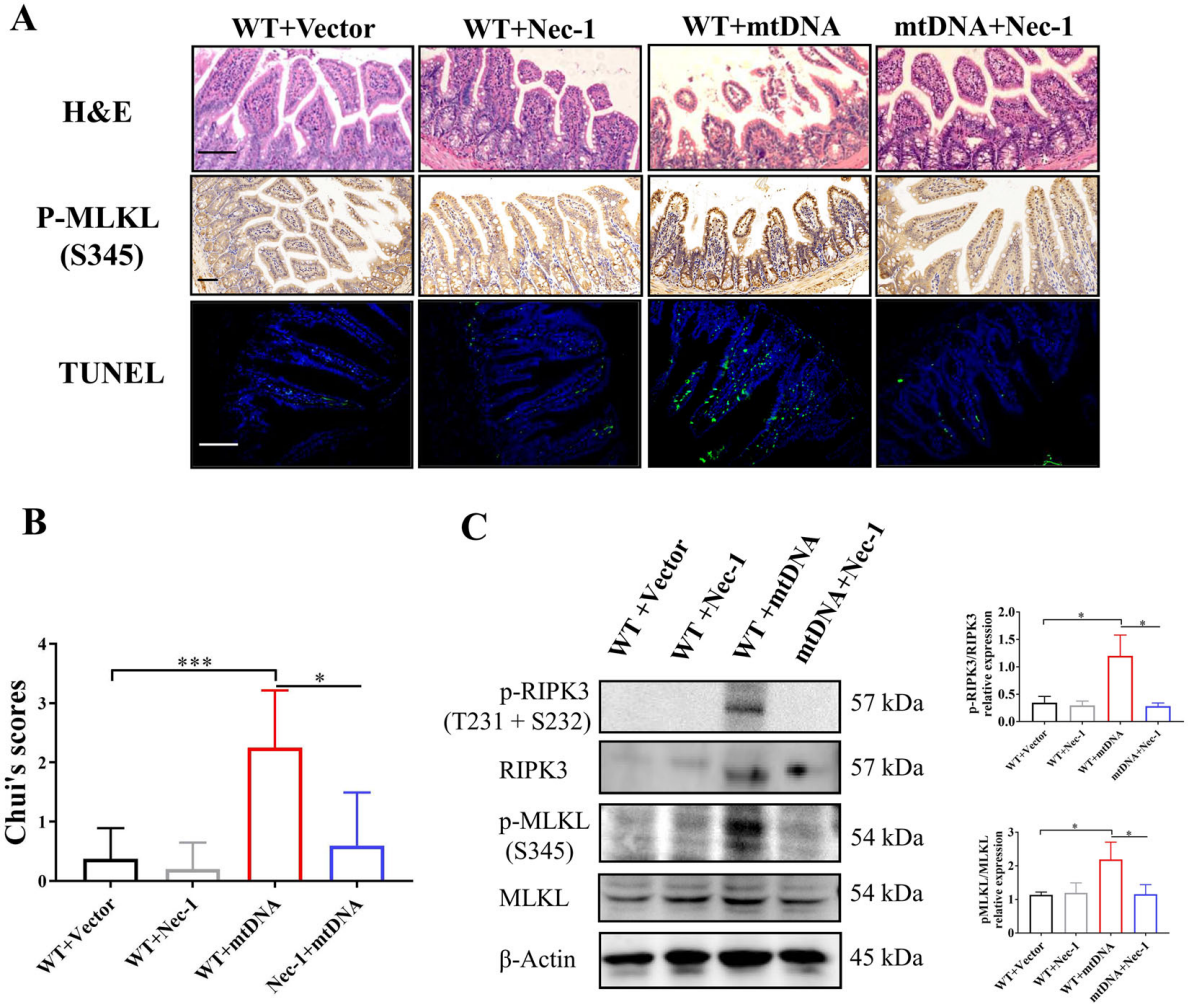
mtDNA contributes to necroptosis-dependent intestinal injury. **A** Representative images of intestinal H&E, IHC (p-MLKL), and TUNEL staining in wild-type mice after the intraperitoneal injection of 5 mg/kg mtDNA or the treatment of nec-1. **B** Intestinal injury was assessed by Chui’s score. **C** Western blot was conducted to analyze necroptosis signaling. Scale bars = 50 μm. Data were shown as the mean ± SD. *P < 0.05, **P < 0.01, ***P < 0.001.

To further investigate the role of mtDNA in necroptosis-mediated intestinal injury, we isolated and purified mtDNA. Mice received an intraperitoneal injection of 5 mg/kg mtDNA. We found that mtDNA induced the destruction of the intestinal mucosa and the increase of TUNEL-positive cells, which were alleviated by the treatment of nec-1 (Fig. 2a, b). Simultaneously, the stimulation of mtDNA also enhanced the levels of p-MLKL in the immunohistochemistry and the western blot (Fig. 2a, c). After the treatment of nec-1, the levels of p-RIPK3 and p-MLKL were decreased (Fig. 2a, c). Therefore, mtDNA promotes intestinal injury by triggering intestinal necroptosis.

### mtDNA-STING signaling promotes intestinal injury through activating necroptosis

**Fig. 3:**
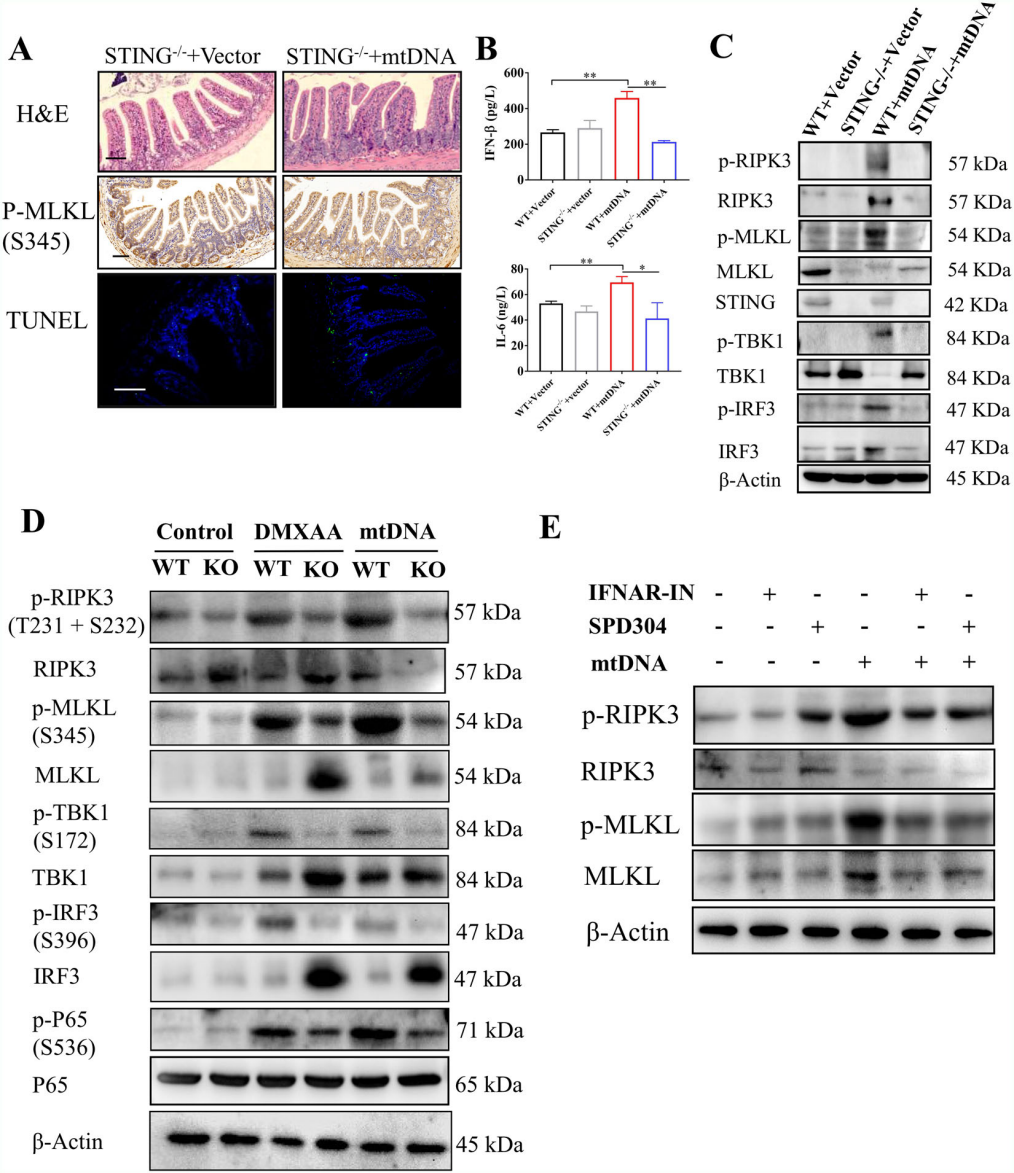
STING signaling promotes mtDNA-mediated necroptosis. (**A**) Representative images of intestinal H&E, IHC (p-MLKL), and TUNEL staining in STING knockout mice after the intraperitoneal injection of mtDNA. (**B**) Plasma IFN-β and IL-6 were tested by ELISA. (**C**) STING signaling and necroptosis were analyzed by western blot. (**D**) Primary intestinal epithelial cells (IECs) isolated from wild-type or STING knockout mice were stimulated with 50 μg/ml DMXAA or 10 μg/ml mtDNA. Western blot was used to analyze STING and necroptosis signaling. (**E**) Primary IECs were treated with 10 μg/ml mtDNA, 1 μM IFN alpha-IFNAR-IN-1 (IFNAR-IN) or 2 μM SPD304. Western blot was used to analyze necroptosis signaling. Scale bars = 50 μm. Data were shown as the mean ± SD. *P < 0.05, **P < 0.01, ***P < 0.001.

mtDNA has been found to be capable of triggering STING signaling^8^. We next sought to explore the role of STING in the mtDNA-mediated intestinal injury. The STING knockout mice were received an intraperitoneal injection of mtDNA. Histopathological evaluation of H&E stained intestines tissue sections showed that STING knockout mice exhibited attenuated intestinal injury compared with wild-type mice after the treatment of mtDNA (Figs. 2a, 3a and Supplementary Fig. 1a). The p-MLKL levels in immunohistochemistry and TUNEL-positive cells were also reduced (Fig. 3a). In addition, the levels of circulating pro-inflammatory cytokines (IFN-β and IL-6) were increased after induction of i.p. injection of mtDNA, which were alleviated by STING depletion (Fig. 3b). As shown by the western blot, the treatment of mtDNA induced significantly increased levels of p-TBK1, p-IRF3, p-RIPK3, and p-MLKL and the decreased levels of STING (Fig. 3c). It has been identified that decreased levels of STING is ascribed to post-activation degradation via autophagy^20^. In comparison, STING depletion reduced not only the levels of p-TBK1 and p-IRF3 but also the levels of p-RIPK3 and p-MLKL (Fig. 3c and Supplementary Fig. 1b). Taken together, necroptosis induced by mtDNA promoted intestinal injury and STING knockout remarkably alleviated intestinal necroptosis and intestinal injury.

As previously mentioned, STING-dependent TBK1 activates both IRF3 and NF-κB, inducing the production of IFN and TNF-α. Moreover, both IFN and TNF-α signaling are capable of triggering necroptosis^14^. Therefore, to determine which downstream signaling of STING leads to necroptosis, primary IECs derived from WT mice or STING knockout mice were isolated and cultured. At first, we tested the effects of STING activated by mtDNA on necroptosis. Primary IECs were treated with STING agonist (DMXAA) and mtDNA. Data of western blot demonstrated that there were increased STING signaling (p-TBK1, p-IRF3, and p-P65) and enhanced necroptosis (p-RIPK3 and p-MLKL) after the stimulation of DMXAA and mtDNA, respectively (Fig. 3d). In comparison, STING depletion significantly attenuated necroptosis (Fig. 3d). We next treated primary IECs with IFN alpha-IFNAR-IN-1 (IFNAR-IN) or SPD304. IFNAR-IN is the inhibitor of the interaction between IFN and IFN receptors and SPD304 is able to block interaction between TNF-αand TNF receptors. After the stimulation of mtDNA, both IFNAR-IN and SPD304 inhibited necroptosis induced by the treatment of mtDNA (Fig. 3e). These data indicate that synergistic IFN and TNF signaling contribute to intestinal necroptosis after mtDNA-mediated activation of STING.

### Necroptosis promotes gut barrier dysfunction following intestinal ischemia reperfusion

**Fig. 4:**
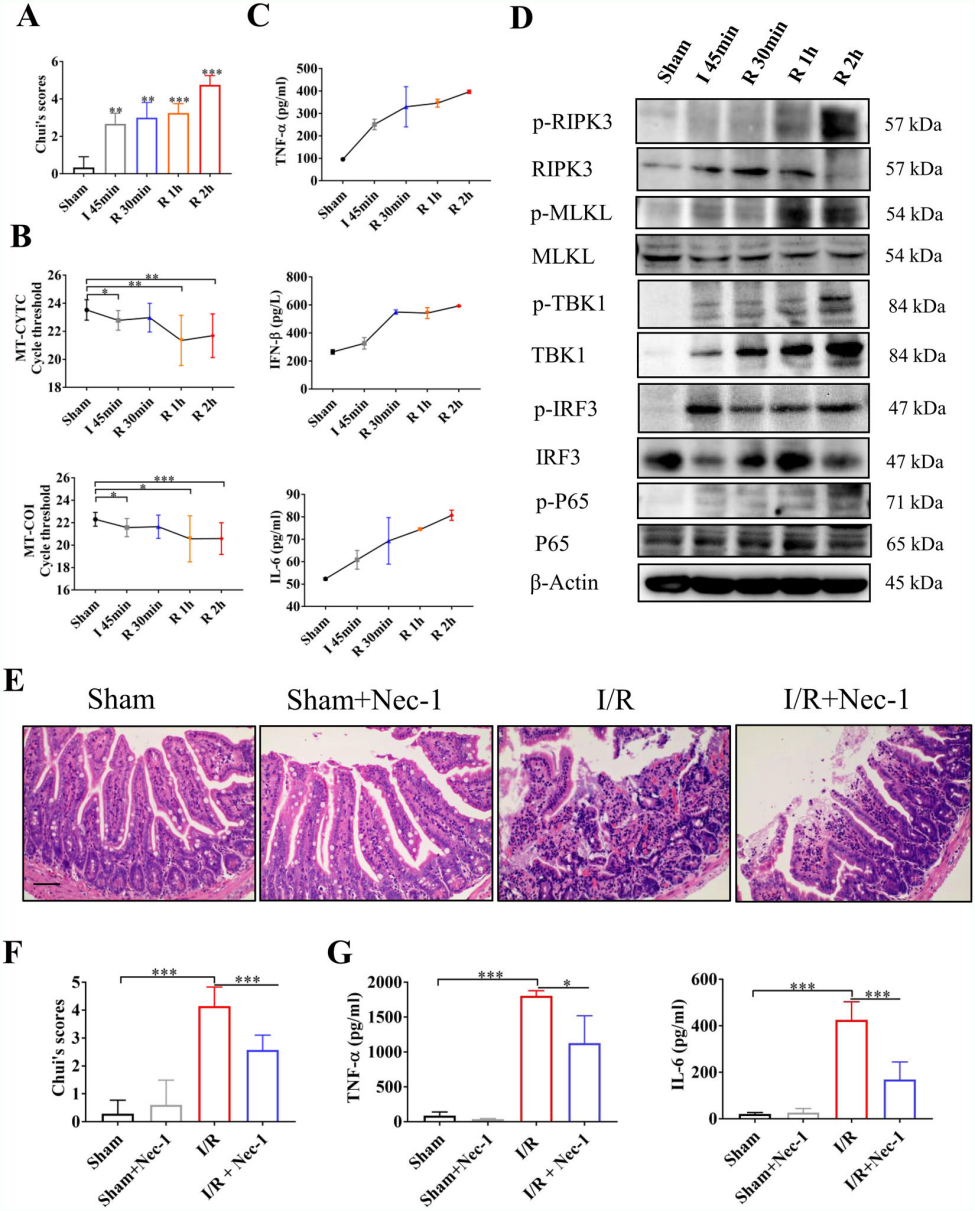
Necroptosis promotes gut barrier dysfunction following intestinal ischemia reperfusion. (**A**) Chui’s score was used to assess intestinal injury for different durations of reperfusion (45 min of ischemia (I 45 min), 30 min of reperfusion (R 30 min), 1 h of reperfusion (R 1 h) and 2 h of reperfusion (R 2 h)). (**B**) mtDNA in the plasma from sham mice and mice with intestinal I/R injury was analyzed via quantitative real-time PCR analysis by amplifying two kinds of mtDNA primers (MT-COI and MT-CYTC). (**C**) Plasma IFN-β, TNF-α, and IL-6 were analyzed by ELISA. (**D**) STING signaling and necroptosis were detected by western blot. (**E**) Representative images of intestinal histology (H&E staining) after intestinal I/R (2 h of reperfusion) or the treatment of necrostatin-1 (nec-1). (**F**) Intestinal injury was assessed by Chui’s score. (**G**) Plasma inflammatory cytokines (TNF-α and Il-6) were detected by ELISA. Scale bars = 50 μm. Data were showed as the mean ± SD. *P < 0.05, **P < 0.01, ***P < 0.001.

As the intestine is vulnerable to ischemic injury, intra-abdominal infection could lead to intestinal I/R injury to a great extent^21,22^. Our previous study has identified the enhanced circulating mtDNA levels in murine intestinal I/R models^6^. The mtDNA concentration in the supernatant was also increased after treating Caco-2 cells with H/R (Supplementary Fig. 2A). To investigate the role of mtDNA-mediated necroptosis in gut barrier dysfunction, we used an intestinal I/R model. To further evaluate the sensitivity of STING signaling and necroptosis during intestinal I/R, we determined the alteration of STING signaling and necroptosis at the different times of intestinal reperfusion. We divided intestinal I/R into 4 groups: 45 min of ischemia (I 45 min), 30 min of reperfusion (R 30 min), 1 h of reperfusion (R 1 h), and 2 h of reperfusion (R 2 h) (Fig. 4a and Supplementary Fig. 3A). After 45 min of ischemia, intestinal injury scores were significantly increased compared with the sham group, and the intestine injury was the most severe at the 2 h of reperfusion among the four groups (Fig. 4a and Supplementary Fig. 4A). DNA in the plasma of mice was extracted and mtDNA levels were evaluated by amplifying two kinds of mtDNA sequences (MT-COI、MT-CYTC) via qPCR. We demonstrated that mtDNA levels were significantly elevated after I 45 min (Fig. 4b). Of note, the circulating pro-inflammatory cytokines (IFN-β, TNF-α, and IL-6) were also enhanced after 45 min of ischemia (Fig. 4c). The results from the western blot exhibited the activation of STING signaling (p-TBK1, p-IRF3, and p-P65) at the early stage of reperfusion and the execution of necroptosis (p-RIPK3 and p-MLKL) at 1 h of reperfusion (Fig. 4d). Therefore, the release of mtDNA is accompanied by the activation of STING, which underlies intestinal necroptosis.

To verify the role of necroptosis in intestinal I/R injury, necrostatin-1 (nec-1), the inhibitor of necroptosis, was administered before intestinal ischemia. There was a significant reduction in histology scores of intestinal injury in the nec-1 treatment group compared with the I/R group after 2 h of reperfusion (Fig. 4e, f). Simultaneously, the administration of nec-1 before intestinal I/R also alleviated TNF-α and IL-6 levels in plasma to some extent (Fig. 4g).

Western blot revealed that intestinal RIPK3 and MLKL were phosphorylated after intestinal 2 h of reperfusion (Fig. 4d and Supplementary Fig. 3B). Interestingly, the total protein levels of RIPK3 were remarkably reduced after intestinal I/R (Fig. 4d and Supplementary Fig. 3B), which was in agreement with the Mangalmurti’s findings^23^. They demonstrated that RBCs induced necroptosis of lung endothelial cells, which resulted in marked loss of RIPK3, and inhibition of necroptosis attenuated the release of RIPK3^23^. Therefore, necroptosis-mediated loss of RIPK3 could contribute to elevated circulating RIPK3. Collectively, these data indicate that necroptosis indeed promotes the intestinal I/R injury.

### STING knockout suppressing intestinal I/R injury

**Fig. 5:**
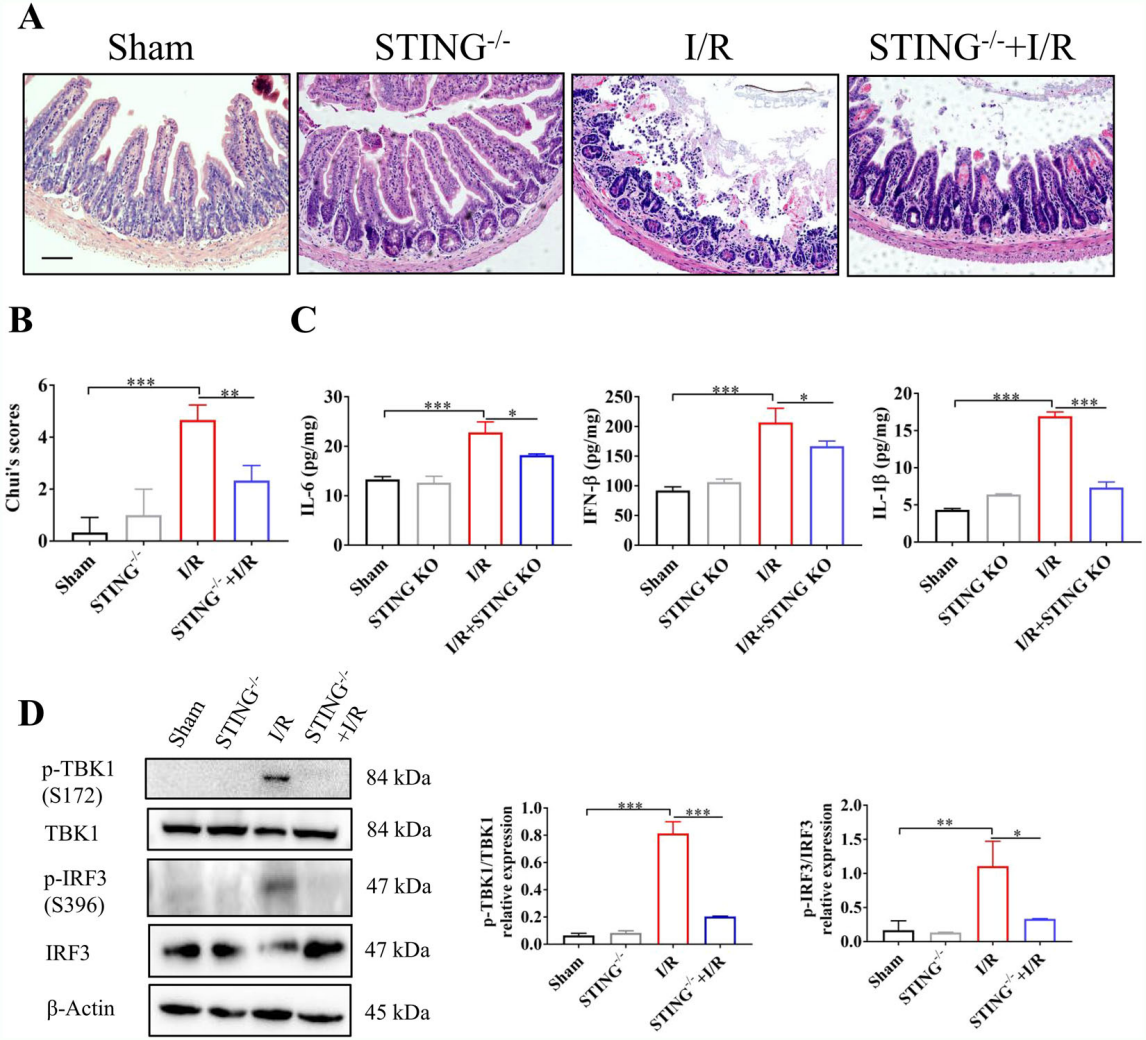
STING knockout alleviates intestinal I/R injury. **(A)** Photomicrographs of intestine in wild-type mice and STING knockout following intestinal I/R injury. **(B)** Intestinal injury was assessed by Chui’s score. **(C)** IFN-β、IL-6 and IL-1βin intestinal tissue were analyzed by ELISA. **(D)** STING signaling was analyzed by western blot. Scale bars = 50μm. Data were showed as the mean ± SD. *P < 0.05, **P < 0.01, ***P < 0.001.

To further confirm the role of STING in intestinal I/R injury, the model of intestinal I/R was used in STING knockout mice. Compared with wild-type mice, STING knockout significantly alleviated intestinal I/R injury (Fig. 5a, b). Meanwhile, STING knockout induced lower production of intestinal inflammatory cytokines (Il-6, IFN-β and IL-1β) after intestinal I/R (Fig. 5c). As shown by the western blot, intestinal I/R induced the downstream activation (p-TBK1 and p-IRF3) of STING (Fig. 5d). STING knockout suppressed phosphorylation levels of TBK1 and IRF3 (Fig. 5d). Simultaneously, intestinal tight junction proteins (ZO-1 and occludin) exhibited an improved appearance in the STING knockout mice (Supplementary Fig. 4A). The above findings indicated that STING signaling may contribute to intestinal injury and an enhanced pro-inflammatory response following intestinal I/R.

### STING deficiency alleviated intestinal necroptosis after intestinal I/R

**Fig. 6:**
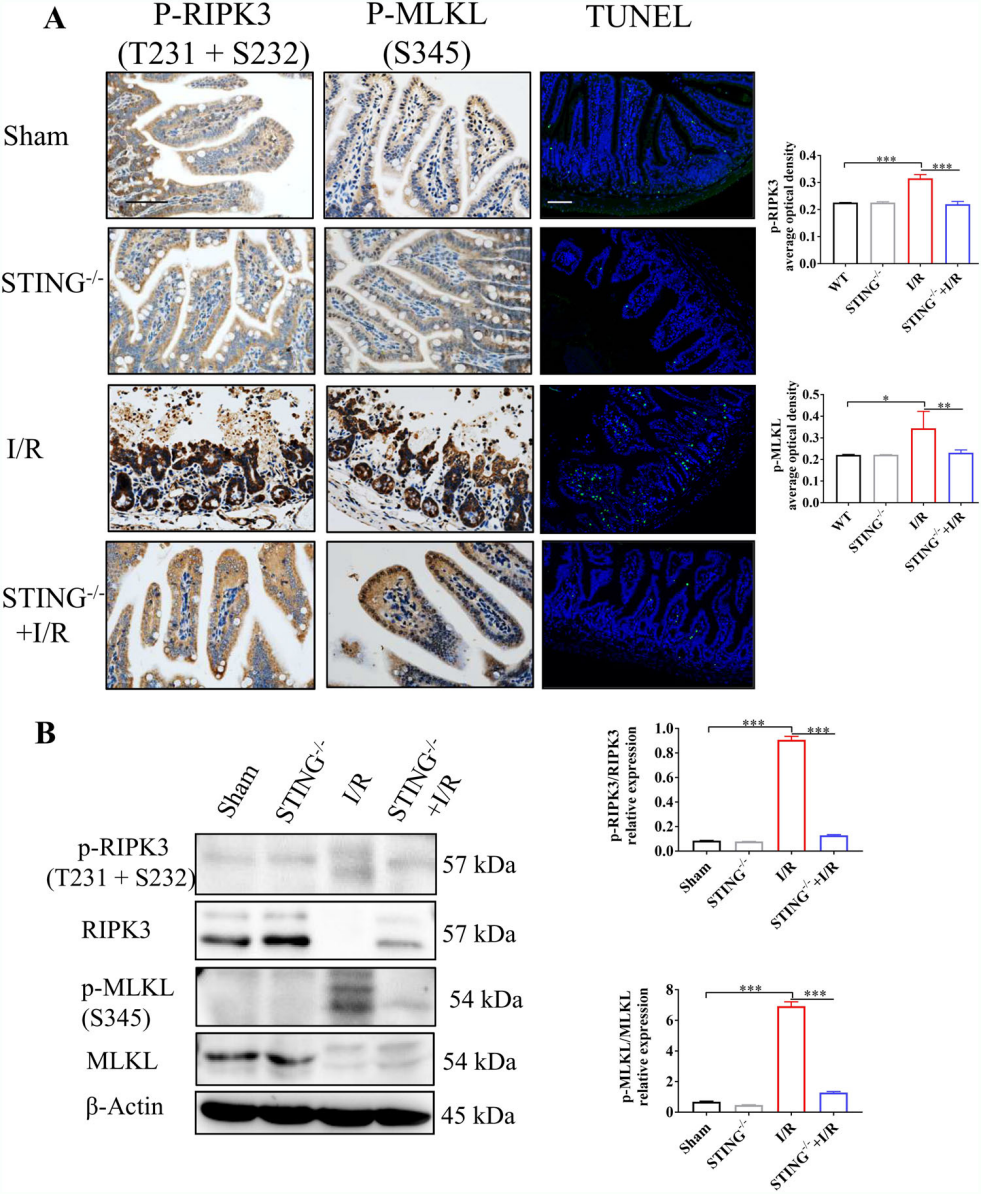
STING knockout attenuates intestinal necroptosis. (**A**–**C**) IHC analysis of p-RIPK3 and p-MLKL in wild-type mice and STING knockout mice following intestinal I/R. TUNEL staining was used to analyze cell death. Image J was used to detect the optical density of p-RIPK3 and p-MLKL in immunohistochemistry. (**B**) Necroptosis signaling was detected by western blot. Scale bars = 50 μm. Data were shown as the mean ± SD. *P < 0.05, **P < 0.01, ***P < 0.001.

We next sought to clarify the role of STING for intestinal necroptosis during intestinal I/R injury. The results from the IHC analysis confirmed that STING knockout significantly decreased the levels of p-MLKL and p-RIPK3 compared with the I/R group (Fig. 6a). Additionally, there was a significant decrease of intestinal TUNEL-positive cells in the STING^-/-^ + I/R group (Fig. 6a). Consistent with the IHC analysis, the western blot also showed the lower production of p-RIPK3 and p-MLKL in the STING^-/-^ + I/R group (Fig. 6b). Taken together, STING knockout suppressed necroptosis in intestinal I/R injury.

## Discussion

In this study, we found that circulating RIPK3 was significantly increased in critically ill patients with intestinal injury. Moreover, circulating RIPK3 had a positive correlation with biomarkers of intestinal injury (I-FABP and D-lactate), which points out a potential role of circulating RIPK3 in indicating intestinal injury. Consistent with the Mangalmurti’s findings^23^, the intestine tissue after 2 h of reperfusion had the lower total protein levels of RIPK3, which could contribute to elevated circulating RIPK3. In contrast, there were significantly increased phosphorylation levels of RIPK3 in intestinal tissue with I/R, indicating that the occurrence of intestinal necroptosis was accompanied by the release of RIPK3.

Mitochondrial DAMPs have been found to trigger inflammatory responses, creating a sepsis-like state after trauma^24^. mtDNA, as one of mitochondrial DAMPs, participates in multiple kinds of innate immune modulation, causing different types of pathologies. Susztak et al.^25^ demonstrated that mtDNA release induced renal inflammation and fibrosis. It was also reported that mtDNA derived from acute liver injury triggered remote lung injury^26^. Our previous studies have identified that mitochondrial oxidative stress-induced mtDNA release after intestinal I/R, subsequently promoting intestinal injury^6^. However, it is unclear how mtDNA triggers intestinal injury during intestinal I/R. In this study, we found circulating RIPK3 had a significant correlation with mtDNA. Moreover, mtDNA has been reported to be capable of activating necroptosis signaling in vitro^17^. Therefore, we speculated that mtDNA release following intestinal I/R activates necroptosis, which promotes intestinal injury. In order to confirm the role of mtDNA for necroptosis in intestinal I/R, we treated mice and primary IECs with mtDNA. Our results indicated that mtDNA indeed induced intestinal necroptosis.

Intestinal I/R injury has been known as the important pathogenesis for acute intestinal barrier disruption. Emerging evidence indicates that STING signaling, as an adaptor for intracellular DNA receptors, is involving in dextran sodium sulfate-induced colitis^11^ and irradiation-mediated intestinal injury^10^. Our group also identified that STING-mediated intestinal barrier disruption resulted in lethal sepsis^12^. However, it remains unclear whether STING signaling contributes to intestinal I/R injury, and how STING signaling affects intestinal I/R injury. Because of STING signaling capable of recognizing mtDNA, we established STING knockout mice to explore the role of STING for mtDNA-mediated necroptosis. We found STING knockout significantly abrogated mtDNA-induced necroptosis and further alleviated intestinal I/R injury. Therefore, STING signaling exerts important roles in the intestinal necroptosis during intestinal I/R injury.

It has been reported the activation of IFN receptors or TNF receptors induces necroptosis^15^. Notably, STING signaling triggers both IRF3 signaling and NF-κB signaling, subsequently promoting the production of IFN and TNF-α. Next, we investigated whether STING activated intestinal necroptosis via IFN or TNF-αsignaling. In primary IECs, the treatment of mtDNA induced necroptosis, which was minimized by STING knockout. After the stimulation of mtDNA, primary IECs were treated with IFNAR-IN or SPD304, respectively. Both IFNAR-IN and SPD304 significantly lowered mtDNA-mediated necroptosis. Hence, STING-dependent both IFN and TNF-αproduction are required for induction of intestinal necroptosis.

Multiple cell death mechanisms including apoptosis^27^, autophagy^28^, ferroptosis^29^, etc, have been reported to be implicated in intestinal I/R injury. It is important to clarify the change of cell death during intestinal I/R injury. Recently, ferroptosis has been found to be involved in intestinal I/R injury and was induced at the early stage of reperfusion^29^. Although Liu et al.^16^ found the existence of necroptosis in intestinal I/R injury, it was unclear how necroptosis changes in intestinal I/R. Our results demonstrated that necroptosis occurred at the later stage of reperfusion. Moreover, along with significant increase of p-RIPK3, the total protein levels of RIPK3 were remarkably reduced, which could contribute to elevated RIPK3 in circulation. However, further studies are needed to clarify the crosstalk among multiple forms of cell death in intestinal I/R injury.

In conclusion, our study reveals that STING signaling is involved in mtDNA-induced necroptosis in intestinal I/R injury. mtDNA induces intestinal necroptosis, further promoting intestinal I/R injury, and STING knockout can ameliorate mtDNA-induced necroptosis in intestinal I/R injury. Furthermore, mtDNA-mediated STING signaling triggers necroptosis through synergistic IFN and TNF-α signaling. In addition, necroptosis-induced RIPK3 release may be as a biomarker of intestinal injury. These findings guide us to extend STING or necroptosis inhibition to intestinal I/R treatment.

## Methods

### Patients

The collection of human blood samples was approved by the Institutional Review Board Ethics Committee at Jinling Hospital (2018NZGKJ-022). Before collecting blood samples, all patients and healthy adult volunteers provided written informed consent. 32 critically ill patients with intra-abdominal infection have been enrolled from December of 2018 to March of 2019. The participants were blinded to the group allocation. Blood samples were collected within one day after admission to the surgical intensive care unit. Then, serum was obtained by centrifuging blood samples at 3000 × *g* at 4 °C for 10 min and stored at -80 °C until further analysis. The acute gastrointestinal injury (AGI) grading system was used to assess intestinal injury of critically ill patients, as previously described^30^. More information of patients can be found in Supplementary Table 1.

### Animals and intestinal I/R model

Male STING knockout (STING^-/-^) and wild-type C57BL/6 J mice (8–12 weeks) were purchased from Model Animals Research Center of Nanjing University. All mice were housed in individual cages with controlled temperature and were fed standard food and water. All mice were acclimated to the environment before use. The care and use of the animals were approved by Jinling Hospital Animal Care Committee.

Entranster^TM^-in vivo (18668-11-2, Engreen Biosystem) was a DNA transfection reagent used to be mixed with mtDNA. For the treatment of mtDNA, mice received an intraperitoneal injection of 5 mg/kg mtDNA, which was extracted from liver tissues by using Mitochondrial DNA Isolation Kit (ab65321, Abcam) and mixed with Entranster^TM^-in vivo. The control group was intraperitoneally injected with Entranster^TM^-in vivo without mtDNA.

Mice were anesthetized intraperitoneally with pentobarbital (50 mg/kg). To establish the experimental intestinal I/R model, a midline laparotomy was conducted to identify the superior mesenteric artery of mice, which was occluded for 45 min by a microvascular clamp and was followed by 0 min, 30 min, 1 h or 2 h of reperfusion. Mice received an intraperitoneal injection of 1.0 mg/kg Necrostatin-1 (nec-1, the inhibitor of necroptosis) (HY-15760, MedChem Express) dissolved in normal saline before the induction of I/R. Mice were randomly divided into three sham groups (8 in each group): (1) wild-type sham group; (2) sham+nec-1 group; (3) STING^-/-^ sham group. I/R groups included I/R group, I/R + nec-1 group, and STING^-/-^ + I/R group.

### Cell hypoxia/reoxygenation, primary IECs isolation, and culture

Caco-2 cells were purchased from Jiangsu KeyGEN BioTECH Corp., Ltd (KG169) and cultured at 37 °C in a 5% CO_2_ humidified incubator. We used Caco-2 cells to establish hypoxia/reoxygenation (H/R) model, which mimic the intestinal I/R injury. In brief, cells were incubated a microaerophilic system (5% CO_2_, 1% O_2_, and 94% N_2_) for 12 h. In order to reoxygenation, cells were then transferred to normoxic conditions for 2 h.

Primary IECs isolation and culture were derived from a protocol adapted from previous studies^31,32^. In brief, the small intestine of mice was dissected out from the duodenum to the ileum and prepared by removing as much adipose tissue as possible, cutting the lumen, and scraping muscle layer. The tissue was washed with ice-cold Mg^2+^- and Ca^2+^-free Hank’s Balanced Salt Solution (HBSS) (KGM24021CS, KeyGEN biotech) containing 100 U penicillin, 100 μg/ml streptomycin (15140163, Thermo Fisher), and 0.5 mM dithiothreitol (DTT) (707265 ML, Thermo Fisher) and then cut into pieces. Tissue was washed five times in the HBSS mentioned above. The tissue was passed over a 40μm cell strainer (15-1040, BIOLOGIX) and remaining tissue was digested in a digestion buffer containing 0.5 mM DTT, 0.1 mg/ml collagenase type XI (C7657, Sigma-Aldrich), and 20 μg/ml dispase II (4942078001, Sigma-Aldrich) in Dulbecco’s Modification of Eagles Medium (DMEM) (10-014-CVR, Corning). The digestion buffer was shaken at 180 rpm in a 37 °C incubator for 30 min. The final digestion mixture was again passed over a 40 μm cell strainer. Next, filter liquor containing isolated intestinal crypts was centrifuged at 300 × *g*, for 4 min, at 4 °C and the remaining sediment was suspended in DMEM containing 100 U penicillin, 100 μg/ml streptomycin, and 2% w/v D-sorbitol (S3889, Sigma-Aldrich). The washing process and resuspended process were repeated for four times. The sediment with crypts was suspended in complete growth media (DMEM, 2.5% v/v FBS (SH30396, HyClone), 100 U penicillin, 100 μg/ml streptomycin, 5 μg/ml transferrin (51300044, Thermo Fisher) and 10 ng/ml epidermal growth factor (SRP3196-500UG, Sigma-Aldrich)). The crypts were cultured at a density of 800 crypts/ml/well in a 6-well culture dish coated by type I collagen (152034, Thermo Fisher) and then incubated at 37 °C in 7.5% CO_2_.

The primary IECs were stimulated with 50 μg/ml DMXAA (HY-10964, MedChem Express), 10 μg/ml mtDNA (extracted from liver tissue by using Mitochondrial DNA Isolation Kit, ab65321, Abcam), 1 μM IFN alpha-IFNAR-IN-1 (HY-111255, MedChem Express) or 2 μM SPD304 (HY-111255, MedChem Express) for 8 h. Mice were pretreated with IFN alpha-IFNAR-IN-1 or SPD304 for 2 h before DMXAA or mtDNA stimulation.

### Histology, immunohistochemistry, and immunofluorescence

For histology, specimens of fresh small intestine were fixed in 4% paraformaldehyde. After dehydrated in ethanol, the intestinal tissue was embedded with paraffin and was stained with hematoxylin and eosin (H&E). To evaluate intestinal injury, Chui’s score system was used as previously described^33^. For immunohistochemistry (IHC), paraffin sections were deparaffinized with xylene and rehydrated with the concentration gradient of ethanol. Then, antigen retrieval was conducted and the samples were incubated with primary antibodies for p-MLKL (phospho S345, ab196436, Abcam) or p-RIPK3 (phospho T231 + S232, ab222320, Abcam) and secondary antibodies (Abcam) and developed with DAB Substrate kit. After visualized with the DAB substrate kit, the sections were rinsed, dehydrated, cleared, and mounted. Finally, the IHC was quantified with Image J software (US National Institutes of Health).

For immunofluorescence staining, frozen sections were cut and mounted on slides. After blocked with 2% bovine serum albumin in PBS at 37 °C for 1 h, the sections were incubated with 1:100 dilutions of primary antibodies, including ZO-1 (61-7300, Invitrogen) and occludin (ab216327, Abcam) and the nuclei were counterstained with DAPI according to the manufacturer’s instructions.

### Terminal deoxynucleotidyl transferase dUTP nick end labeling staining

Programmed cell death in the intestinal epithelium was detected by terminal deoxynucleotidyl transferase dUTP nick end labeling (TUNEL) staining using a commercial kit (KGA7072, KeyGEN biotech) according to the manufacturer’s instructions.

### Western blot

Proteins from the intestinal tissue or the primary IECs were separated by 10% SDS-PAGE and transferred onto polyvinylidene difluoride (PVDF) membranes. The PVDF membranes were incubated overnight at 4°C with primary antibodies, including MLKL (A5579, Abclonal), p-MLKL (phospho S345, ab196436, Abcam), RIPK3 (17563-1-AP, Proteintech), p-RIPK3 (phospho T231 + S232, ab222320, Abcam), STING (19851-1-AP, Proteintech), TBK1 (38066, Cell Signaling), p-TBK1 (phospho S172, 5483, Cell Signaling), IRF3 (4302, Cell Signaling), p-IRF3 (phospho S396, 29047, Cell Signaling), P65 (A2547, Abclonal), p-P65 (phospho S536, AP0475, Abclonal) and β-actin (4970, Cell Signaling). Proteins in western blot were quantified in optical density units via Image J software.

### Quantitative real-time PCR analysis

DNA in plasma, serum or cell culture supernatant was extracted by using commercially available kits (QIAamp DNA Mini kit, 51304, QIAGEN) according to the manufacturer’s instructions. Then, real-time PCR was performed by using SYBR Green qPCR Master Mix (Q711-02/03, Vazyme biotech). The relevant sequences of primers for qPCR analyses are shown in Supplementary Table 2. Of note, higher cycle thresholds represent lower levels of mtDNA.

### Enzyme-linked immunosorbent assay (ELISA)

The protein levels of human RIPK3 (HM11309, Bio-Swamp), human I-FABP (HM10888, Bio-Swamp), human IL-6 (KGEHC007, KeyGEN biotech), human D-lactate (K667-100, BioVision), murine TNF-α (ANG-E21030M, AngleGene, Nanjing, China), murine IL-6 (KGEMC004, KeyGEN biotech), murine IFN-β (ANG-E21047M, AngleGene, Nanjing, China) and murine IL-1β (ANG-E21583M, AngleGene, Nanjing, China) in the plasma, serum or intestinal tissue were evaluated by using commercially available ELISA kits according to the manufacturer’s instructions.

### Statistical analysis

The related results were shown as the mean ± standard deviation (SD) or median with interquartile range (IQR), as appropriate. Differences between groups were compared by the two-tailed student’s t-test or one-way analysis of variance. At least three independent experiments were conducted to confirm the results. Statistical analyses were conducted by using GraphPad Prism software 7.0. *P* < 0.05 was considered for statistical significance (* < 0.05; ** < 0.01; *** < 0.001).

## Supplementary information

Supplementary figure lengends

Supplementary Table 1

Supplementary Table 2

Supplementary fig1

Supplementary fig 2

Supplementary fig 3

Supplementary fig 4
