# Supplementary material for: mtDNA-STING pathway promotes necroptosis-dependent enterocyte injury in intestinal ischemia reperfusion
Source: Cell Death Dis. 2020 Dec 11;11(12):1050. doi: 10.1038/s41419-020-03239-6 (PMC7732985; doi:10.1038/s41419-020-03239-6)
Supplement: Supplementary file 1 — Supplementary figure lengends [file 41419_2020_3239_MOESM1_ESM.docx]

**Supplementary Figure 1** STING knockout alleviates mtDNA-induced intestinal injury and necroptosis. (A) Intestinal injury was assessed by Chui’s score. (B) Image J was used to detect optical density of p-RIPK3 and p-MLKL in the western blot. Data were showed as the mean ± SD. ^⁎^P＜0.05, ^⁎⁎^P＜0.01, ^⁎⁎⁎^P＜0.001.

**Supplementary Figure 2** There is increased mtDNA concentration in the supernatant of H/R-treated Caco-2 cells. (A) mtDNA in the supernatant of H/R-treated Caco-2 cells was analyzed via quantitative real-time PCR analysis by amplifying one kind of mtDNA primers (MT- COX1). Data were showed as the mean ± SD. ^⁎^P＜0.05, ^⁎⁎^P＜0.01, ^⁎⁎⁎^P＜0.001.

**Supplementary Figure 3** Different durations of reperfusion in intestinal I/R injury. (A) Photomicrographs of intestine in wild-type mice following different durations of reperfusion (45min of ischemia (I 45 min), 30min of reperfusion (R 30 min), 1 h of reperfusion (R 1h) and 2h of reperfusion (R 2h)). (B) Activation of necroptosis in intestine following intestinal I/R was analyzed by western blot. Scale bars=50μm. Data were showed as the mean ± SD. ^⁎^P＜0.05, ^⁎⁎^P＜0.01, ^⁎⁎⁎^P＜0.001.

**Supplementary Figure 4** STING knockout restores intestinal tight junction proteins after intestinal I/R injury. (A) Intestinal tight junction proteins (ZO-1 and occludin) were determined by immunofluorescence. Nuclei were counterstained with DAPI. Scale bars=50μm.
