# Supplementary material for: mtDNA-STING pathway promotes necroptosis-dependent enterocyte injury in intestinal ischemia reperfusion
Source: Cell Death Dis. 2020 Dec 11;11(12):1050. doi: 10.1038/s41419-020-03239-6 (PMC7732985; doi:10.1038/s41419-020-03239-6)
Supplement: Supplementary file 2 — Supplementary Table 1 [file 41419_2020_3239_MOESM2_ESM.docx]

Supplementary Table 1

| **Characteristics** | **Number** |
| --- | --- |
| **Total patients** | 32 |
| **Gender (male), n (%)** | 24 (75.00) |
| **Age, median (IQR), y** | 61.50 (43.00-67.00) |
| **BMI, mean (SD)** | 23.76 (19.43-25.71) |
| **SOFA score, median (IQR)** | 5.5 (1-9.75) |
| **APACHE II score, median (IQR)** | 11.00 (7.25-17.00) |
| **Primary Disease, n (%)** |  |
| Trauma | 8 (25.0) |
| Surgical complication^a^ | 20 (62.5) |
| Spontaneous gastrointestinal perforation | 2 (6.3) |
| Others^b^ | 2 (6.3) |
| **Abdominal viscera injurie (%)** |  |
| Stomach | 6 (18.8) |
| small intestine | 13 (40.6) |
| vermiform appendix | 5 (15.6) |
| Colon | 2 (6.3) |
| Multiple viscera | 6 (18.8) |
| **Death, n (%)** | 11 (34.38) |

^a^Patients who developed into intestinal fistula after abaominal surgery were defined as surgical complication

^b^Two cases of acute suppurative appendicitis
