# Supplementary material for: mtDNA-STING pathway promotes necroptosis-dependent enterocyte injury in intestinal ischemia reperfusion
Source: Cell Death Dis. 2020 Dec 11;11(12):1050. doi: 10.1038/s41419-020-03239-6 (PMC7732985; doi:10.1038/s41419-020-03239-6)
Supplement: Supplementary file 3 — Supplementary Table 2 [file 41419_2020_3239_MOESM3_ESM.docx]

Supplementary Table 2

| **mtDNA sequence** | **Forward (F) and reverse (R) primer** |
| --- | --- |
| COI-F (mouse) | GCCCCAGATATAGCATTCCC |
| COI-R | GTTCATCCTGTTCCTGCTCC |
| COX1-F (human) | TCATCTGTAGGCTCATTC |
| COX1-R | GCGATCCATATAGTCACT |
| ND1-F (human) | GCTACGACCAACTCATAC |
| ND1-R | GAATGCTGGAGATTGTAATG |
| ND2-F (human) | CACAGAAGCTGCCATCAAGTA |
| ND2-R | CCGGAGAGTATATTGTTGAAGAG |
| CYTC-F (mouse)  CYTC-R | GACTTGCAACCCTACACGGAT  CCGGTTAGACCACCAACTGT |
